# Influence of Root Canal Curvature on the Accuracy of Root ZX Electronic Foramen Locator: An *In Vitro* Study

**DOI:** 10.22037/iej.2017.34

**Published:** 2017

**Authors:** Masoud Saatchi, Shiva Iravani, Mehrdad Akhavan Khaleghi, Amin Mortaheb

**Affiliations:** a *Dental Research Center, Department of Endodontics, Dental School, Isfahan University of Medical Sciences, Isfahan, Iran; *; b *Department of Endodontics, Dental School, Ahvaz Jundishapour University of Medical Sciences, Ahvaz, Iran;*; c * Department of Prosthodontics, Dental School, Isfahan University of Medical Sciences, Isfahan, Iran; *; d * Department of Endodontics, Dental School, Islamic Azad University of Khorasgan, Isfahan, Iran*

**Keywords:** Accuracy, Curved Root Canals, Electronic Apex Locator, Working Length

## Abstract

**Introduction:**

The aim of this *in vitro* study was to evaluate the correlation between accuracy of Root ZX electronic foramen locator and root canal curvature.

**Methods and Materials:**

One hundred and ten extracted mandibular molars were selected. Access cavity was prepared and coronal enlargement of mesiobuccal canal was performed. A #10 Flexofile was inserted into the mesiobuccal canal, and a radiography was taken to measure the degree of curvature by Schneider's method. The actual working length (AWL) was defined by inserting the file until its tip could be observed at a place tangential to the major apical foramen and then 0.5 mm was subtracted from this measurement. For the electronic working length (EWL) measurement, the apical 3 or 4 mm of the root was embedded in alginate as the electrolyte material. The file was inserted into the root canal to the major foramen, until the APEX reading was shown on the electronic device and then pulled back until the visual display showed the 0.5-mm mark. The AWL was subtracted from the EWL to define the distance between the file tip and the point 0.5 mm coronal to the major apical foramen. Data were analyzed using the Pearson’s correlation coefficient.

**Results:**

The accuracy of Root ZX within ±0.1 mm and ±0.5 mm was 38.2% and 94.6%, respectively. There was no correlation between the distance from the EWL to the AWL and the degree of root canal curvature (r=0.097, *P*=0.317).

**Conclusion:**

Root canal curvature did not influence the accuracy of Root ZX foramen locator.

## Introduction

An ideal root canal treatment should be limited to the root canal system. Any procedure beyond or less than this point may increase the risk of treatment failure [1]. As a result, working length (WL) determination is a crucial factor in successful root canal therapy. The apical constriction (AC) is suggested as the end-point of root canal treatment [2]. This anatomical landmark is a point where pulpal and periodontal tissues reach together and is identified as minor apical foramen [3]. It is generally accepted to be located at 0.5-1 mm coronal to the radiographic apex [2]. However, Dummer *et al.* [3] reported that AC might be located on one side of root at a distance up to 3 mm from the anatomical apex. Moreover, the position and topography of minor foramen varies between teeth, making it difficult to determine clinically [3]. 

Radiography has been routinely used for WL determination. This method is influenced by limitations such as file size, film position, image distortion, image magnification, tooth inclination, superimposition of bony structures and interpretation variability, resulting in inaccurate findings [4-6]. Furthermore, radiographies show two dimensions of a three-dimensional structure, which might result in loss of data in some cases [4, 5, 7].

Electronic foramen locators (EFLs) were designed to overcome the limitations of radiographs. Sunada [8] was the first to introduce EFLs in clinical practice. Initial devices determined WL by calculating electrical resistance between the periodontal ligament and oral mucosa, which is the constant value of 6.5 kΩ. The first EFLs did not exhibit sufficient accuracy for measuring the WL and were influenced by various root canal irrigation solutions. The subsequent EFLs have overcome this problem and are capable of measuring the canal length in the presence of electrolytes [9]. The Root ZX (J. Morita Corp, Tokyo, Japan) measures the impedance ratio of two different frequencies (0.4 and 8 kHz) for determining the tip of the file in the canal, regardless of the type of electrolyte, and requires no calibration [6, 10]. The accuracy of this device between the *in vivo* and *in vitro* models is not different [11]. 

The influence of various factors such as EFL type [12], tooth type [13], tooth length [14], apical foramen diameter [15], device generation [16], pulp vitality [17], apical periodontitis [18], irrigation solution [19] endodontic perforation [20] and on the accuracy of EFLs has been evaluated. Root canal curvature, as an anatomical factor, may also influence the accuracy of EFLs. To date, there have been only a few and controversial reports on this issue [21-23]. Thus, the aim of this *in vitro* study was to investigate the possible correlation between accuracy of Root ZX foramen locator and degree of root canal curvature in mesiobuccal canals of mandibular molars.

## Materials and Methods

A total of 110 extracted human mandibular molars were selected. The teeth were kept in 5.25% sodium hypochlorite for 3 h and then rinsed with tap water. Residual soft tissue and calculus were removed from the root surface using periodontal scalers and curettes. The teeth were evaluated for root cracks or fractures, root resorptions, open apices, restorations and previous root canal treatment; teeth with any of these characteristics were excluded. Access cavity preparation was accomplished using a #1014 round diamond bur (KG, Sorensen, Sao Paulo, SP, Brazil) and finished with an Endo Z bur (Dentsply Maillefer, Ballaigues, Switzerland) under cool water spray. The mesiobuccal root canal was evaluated. The mesiobuccal cusp was ground to provide a stable reference point using the same bur. Pulp tissue remnants were removed with #10 and #15 Flexofile (Dentsply-Maillefer, Ballaigues, Switzerland). Coronal enlargement of the root canal was carried out by #1, 2 and 3 Gates-Glidden drills (Mani, Tochigi, Japan). The root canal was irrigated with 2.5% sodium hypochlorite using a 27-gauge needle after each instrument.

For root canal curvature measurement, the tooth was mounted in a plaster block in order to facilitate radiography in buccolingual direction except for the crown and the apical 3-4 mm of the roots. A #10 Flexofile was inserted into the mesiobuccal canal until the tip of the file could be observed through the major apical foramen. Then the canal curvature was measured based on Schneider's method using a digital radiograph [24]. Electronic and actual working lengths were measured by an experienced endodontist similar to the previous study [18].

**Figure 1:**
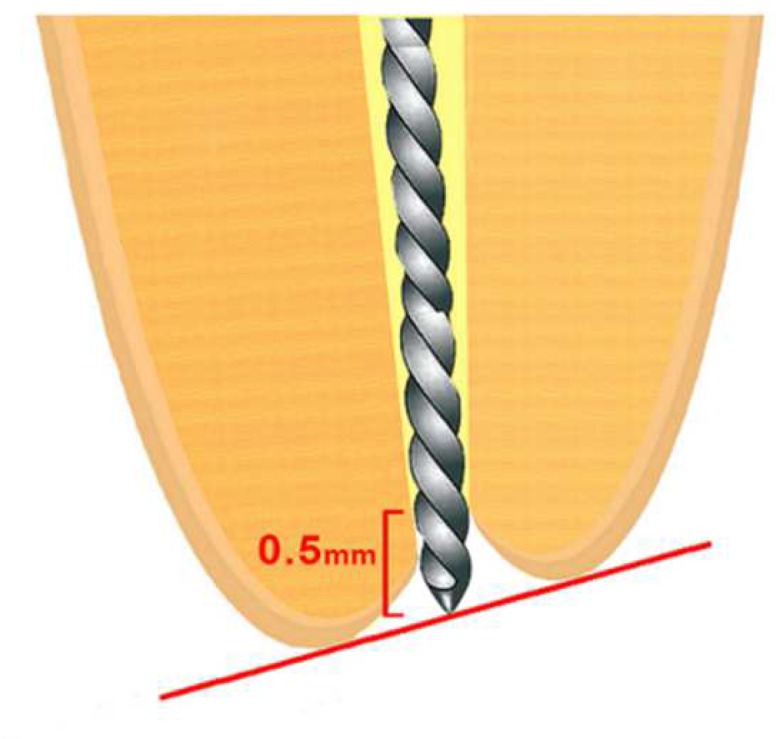
Actual working length measurement. The tip of the file was inserted into the root canal and placed tangential to the major apical foramen. The apical landmark was considered at 0.5 mm coronal to the position

For actual WL (AWL) measurement, a #10 Flexofile was inserted into the mesiobuccal canal until its tip could be observed through the major apical foramen under ×16 magnification by using a dental operating microscope (OPMI Primo, Carl Zeiss, Germany). The file was then pulled back until its tip was placed tangential to the major apical foramen (Figure 1). A silicone stop was placed to the ground mesiobuccal cusp, which was selected as a coronal reference point; the file was removed from the root canal and the distance between its tip and the silicone stop was measured with a high-precision digital caliper (Mitutoyo Corp, Tokyo, Japan). Then 0.5 mm was subtracted from this measurement. The measurements were repeated 3 times and the mean of the values was recorded as the AWL.

For electronic WL (EWL) measurement, Root ZX (J. Morita Corp, Tokyo, Japan) was used according to the manufacturer’s instructions. The tooth was fixed in a plate containing an electrolyte and 3-4 mm of the root end was placed completely in the electrolyte. Alginate (Alginoplast; Heraeus-Kulzer, Hanau, Germany) was used as the electrolyte material. The excess irrigation solution was removed from the pulp chamber using a cotton pellet. The lip clip was placed in the plate in contact with alginate. A #10 Flexofile was then connected to the file clip of the device and the file was gently inserted apically into the canal using watch-winding motions until the visual display showed the “apex mark” and then pulled back until the display indicated the 0.5-mm mark. The measurement was considered correct if the reading remained stable for at least 5 sec. A silicone stop was placed at the reference point; the file was removed from the root canal and the distance between its tip and the silicone stop was measured with the same digital caliper. The operator repeated the measurements 3 times and the mean of the values was recorded as EWL.

**Table 1.** Number (percent) of the differences between EWL and AWL

**EWL-AWL**	**Number (%)**
**> 0.5**	2 (1.8)
**0.1 to 0.5**	51 (46.4)
**-0.1 to 0.1**	42 (38.2)
**-0.5 to -0.1**	11 (10.0)
**<-0.5**	4 (3.5)

**Table 2:** Degrees of root canal curvature and distances from the file tip (EWL) to the point 0.5 mm coronal to the major foramen (mm)*

**Tooth**	**Curvature**	**Difference**	**Tooth**	**Curvature**	**Difference**	**Tooth**	**Curvature**	**Difference**
**1**	27	0.00	**38**	35	-0.06	**75**	32	-0.07
**2**	27	-0.07	**39**	29	-0.06	**76**	21	0.00
**3**	31	-0.03	**40**	21	-0.04	**77**	22	0.04
**4**	25	-0.40	**41**	24	-0.06	**78**	41	-0.24
**5**	23	0.13	**42**	17	-0.04	**79**	35	-0.06
**6**	28	0.03	**43**	31	0.00	**80**	36	0.00
**7**	35	-0.10	**44**	30	-0.07	**81**	53	0.03
**8**	29	0.07	**45**	27	0.00	**82**	41	0.06
**9**	37	0.00	**46**	33	0.04	**83**	17	-0.07
**10**	27	0.00	**47**	55	-0.10	**84**	36	-0.03
**11**	55	-0.13	**48**	21	-0.20	**85**	17	-0.13
**12**	29	0.00	**49**	37	0.00	**86**	26	0.04
**13**	12	0.00	**50**	10	-0.30	**87**	24	-0.03
**14**	32	-0.03	**51**	25	-0.16	**88**	17	-0.10
**15**	38	0.00	**52**	18	0.03	**89**	32	0.00
**16**	58	0.07	**53**	34	0.00	**90**	30	0.00
**17**	31	-0.06	**54**	20	-0.10	**91**	27	0.00
**18**	20	-0.33	**55**	23	-0.36	**92**	30	0.10
**19**	36	-0.03	**56**	9	-0.03	**93**	51	-0.33
**20**	31	-0.40	**57**	37	0.00	**94**	15	0.00
**21**	25	-0.06	**58**	23	-0.16	**95**	41	0.00
**22**	28	0.00	**59**	27	-0.10	**96**	50	0.00
**23**	17	0.44	**60**	22	-0.13	**97**	38	0.00
**24**	30	0.00	**61**	16	0.00	**98**	32	0.00
**25**	37	0.00	**62**	21	0.13	**99**	33	0.17
**26**	39	-0.03	**63**	28	0.00	**100**	29	-0.06
**27**	21	0.00	**64**	36	0.00	**101**	18	0.00
**28**	29	-0.20	**65**	13	0.13	**102**	36	-0.30
**29**	22	0.00	**66**	22	-0.06	**103**	35	-0.04
**30**	31	0.00	**67**	22	0.00	**104**	21	-0.30
**31**	34	-0.60	**68**	32	0.00	**105**	37	-0.16
**32**	27	-0.07	**69**	12	-0.26	**106**	29	-0.10
**33**	41	0.10	**70**	28	0.14	**107**	43	0.00
**34**	28	-0.04	**71**	36	0.07	**108**	33	0.10
**35**	33	-0.17	**72**	42	-0.30	**109**	32	-0.50
**36**	39	0.00	**73**	28	-0.04	**110**	28	0.03
**37**	35	-0.10	**74**	24	-0.04			

* Negative numbers indicated measurements short of the AWL (under) and positive numbers indicated measurements; exceeding the AWL (over)

In each tooth, the AWL was then subtracted from the EWL to define the distance between the tip of the file (EWL) and the point 0.5 mm coronal to the major apical foramen (AWL). Positive numbers indicated measurements exceeding the AWL (over) and negative numbers indicated measurements short of the AWL (under). Data were then subjected to statistical analysis using SPSS 18 (SPSS Inc, Chicago, IL, USA). The correlation between the distance from the file tip (EWL) to the point 0.5 mm coronal to the major apical foramen (AWL) and the degree of root canal curvature was evaluated using Pearson’s correlation coefficient. Correlation was significant at the 0.01 level. 

## Results

**Figure 2:**
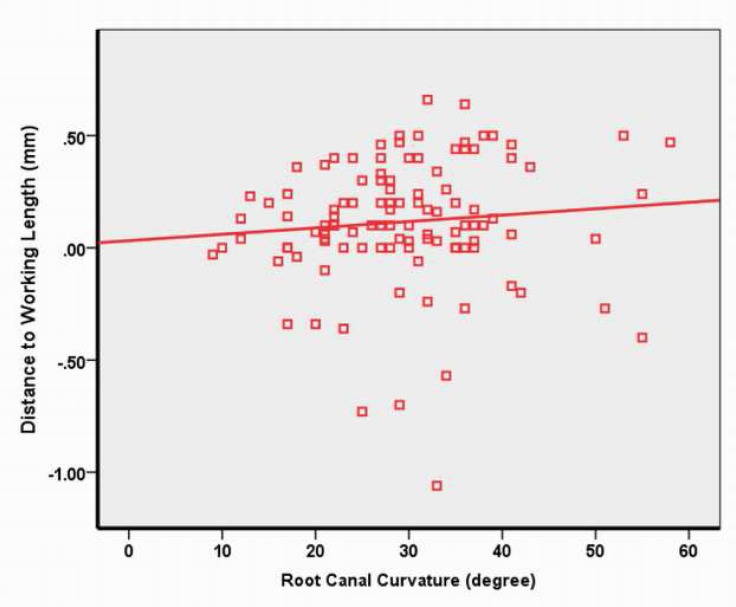
Correlation between the distance from the file tip (EWL) to the point 0.5 mm coronal to the major foramen (AWL) and the degree of root canal curvature

In the present study, 110 mesiobuccal root canals of mandibular molars with root canal curvatures between 9 and 58 degrees were evaluated. Degrees of root canal curvature and distances from the file tip (EWL) to the point 0.5 mm coronal to the major foramen (AWL) is shown in Table 1. Table 2 shows the position of the file tip as determined electronically relative to the AWL. The accuracy of Root ZX within the error range of ±0.1 mm and ±0.5 mm was 38.2% and 94.6%, respectively. Figure 2 presents a scatter plot of the correlation between the distance from the file tip (EWL) to the point 0.5 mm coronal to the major foramen (AWL) and the degree of root canal curvature. There was no significant correlation between the distance from the EWL to the AWL and the degree of root canal curvature (r=0.097, *P*=0.317).

## Discussion

In this *in vitro* study the correlation between accuracy of Root ZX electronic foramen locator and degree of root canal curvature in mesiobuccal canals of mandibular molars was evaluated. Although there is a poor correlation between accuracy of Root ZX electronic foramen locator and root canal curvature, this correlation is not statistically significant.

The accuracy of frequency-dependent EFLs has been usually reported with an error range of ±0.5 mm, which is approximately 65-100% [25]. In this study, the percentage for this range was 94.6, which shows a high accuracy for the Root ZX device. However, this percentage decreased to 38.2 within the error range of ±0.01 mm. 

The accuracy of EFLs has been reported to improve by elimination of coronal file interference within the root canal space [26]. In addition, the accuracy of Root ZX in shorter teeth is higher than that in longer ones because the file interference within the root canal space in short teeth is less than that in long ones [14]. Herrera *et al.* [27] claimed that the accuracy of EFLs might be influenced by file size as smaller files leave some space within the root canal whereas larger files fit more tightly. Thus, file interference and constraint within the root canal space may affect the accuracy of EFLs. In this study, it was assumed that by increasing the root canal curvature the interference of file with dentinal walls would increase, possibly influencing the readings of EFLs. Therefore, the aim of this study was to evaluate the effect of root canal curvature on the accuracy of a well-known EFL, the Root ZX.

A few studies evaluated the effect of root canal curvature on the accuracy of EFLs. Sadeghi *et al.* [21] compared 20 maxillary centrals with straight canals and 20 mesiobuccal canals of mandibular molars with a curvature range of 25-30 degrees and reported that the accuracy of Raypex 5 foramen locator in the straight root canal of maxillary centrals was more than that in curved canals of mandibular molars. Santhosh *et al.* [22] divided 60 mesiobuccal canals of mandibular molars into 3 groups of mild, moderate and severe curvatures. They reported that the accuracy of Root ZX in the mildly curved canals was more than that in the moderately and severely curved canals. Tian *et al.* [23] evaluated 123 root canals, divided them into 3 groups of mild, moderate and severe curvatures, and concluded that root canal curvature has no influence on the accuracy of the EFLs. However, in the present study, 110 mesiobuccal canals of mandibular molars with a curvature range of 9‒58 degrees were investigated using Root ZX and no correlation was found between accuracy of the EFL and the degree of root canal curvature.

Differences between the results of different studies may be explained by differences in tooth type, device type, study design, sample size and data analysis.

Root canal preparation should be limited to the canal terminus, which is considered by most clinicians as the minor apical foramen or the apical constriction. It is located at 0.5-1 mm coronal to the major apical foramen [2]. Therefore, the apical landmark was considered at 0.5 mm coronal to the major apical foramen. Obviously, EFLs are not capable of detecting the apical constriction and root apex but they detect the major apical foramen [28]. However, they are generally called “electronic apex locator”. Therefore, the use of an “electronic root canal length measurement device”, “electronic apical foramen locators” or simply “EFL” may be more meaningful [14, 18]. The devices are sometimes classified by *generation*, which is not helpful to clinicians. In addition, the information provided by manufacturers is often too limited to make it possible to classify them and thus it is better suited for marketing issues [29, 30].

Alginate mass is a useful tool in evaluating the performance of EFLs [31]. Hence, alginate was used as the embedding medium. In addition, 2.5% sodium hypochlorite was used as an irrigation solution. Evidence showed that the accuracy of Root ZX was not influenced by the solution [32]. 

The root canal length measurement using radiography becomes more complicated as the degree of root curvature increases [33]. For roots with buccal or lingual curvatures, such as buccal curvature in the palatal roots of maxillary molars, the capability of a dentist to measure the working length using radiography might be impaired [34]. Therefore, considering the results of the present study, it might be more helpful for clinicians to use an EFL to determine the working length in curved root canals.

## Conclusion

The *in vitro* accuracy of Root ZX foramen locator was not influenced by the root canal curvature.
